# Management of Fatigue in Patients with Advanced Cancer

**DOI:** 10.1007/s11864-022-01045-0

**Published:** 2023-01-19

**Authors:** Patrick Stone, Diego Ezequiel Candelmi, Kerran Kandola, Ligia Montero, Dom Smetham, Sahil Suleman, Asanga Fernando, Rocío Rojí

**Affiliations:** 1 Marie Curie Palliative Care Research Department, Division of Psychiatry, University College London (UCL), 6th Floor, Maple House, 149 Tottenham Court Road, W1T 7NF, London, UK; 2 Paliative Medicine Department, Clinica Universidad de Navarra, Pamplona, Spain; 3 departametno de Oncología de la Clinica Universidad de Navarra, Pamplona, Spain; 4 Cancer Psychological Support Team, St George’s University Hospitals NHS Foundation Trust, London, UK; 5 Cancer Psychological Support Team, Department of Medical Oncology and Department of Liaison Psychiatry, St George’s University Hospitals NHS Foundation Trust, London, UK; 6 Instituto de Investigación Sanitaria de Navarra (IdiSNA), Navarra, Spain

**Keywords:** Fatigue, Neoplasm, Palliative care, Therapeutics, Exercise, Psychotherapy

## Abstract

Fatigue is a common and distressing symptom experienced by patients with cancer. It is most common in patients with locally advanced or metastatic incurable disease. It can have profound effects on quality-of-life and physical functioning. In addition to general supportive measures (directed at tackling contributory conditions and comorbidities), a variety of specific interventions have been developed which can be broadly categorised as physical therapies, psychological therapies or medication. There is some evidence that each of these approaches can have benefits in patients with earlier stage disease, those undergoing active treatment and in cancer survivors. The best evidence is for aerobic exercise, yoga, cognitive-behavioural therapy (CBT) and psycho-educational interventions. Less strong evidence supports the use of medications such as methylphenidate or ginseng. In patients with advanced disease, it is likely that the mechanisms of fatigue or the factors contributing to fatigue maintenance may be different. Relatively fewer studies have been undertaken in this group and the evidence is correspondingly weaker. The authors recommend the cautious use of aerobic exercise (e.g. walking) in those who are still mobile. The authors advise considering the use of psycho-educational approaches or CBT in those patients who are able to engage in such forms of therapy. In patients near the end-of-life, the authors advise use of dexamethasone (short-term use) and other pharmacological treatments only on the basis of a clinical trial.

## Introduction

Cancer-related fatigue (CRF) is a debilitating symptom that can affect patients throughout their illness. The National Comprehensive Cancer Network has defined CRF as “a distressing, persistent, subjective sense of physical, emotional, and /or cognitive tiredness or exhaustion related to cancer or cancer related treatment that is not proportional to recent activity and interferes with usual functioning” (1••). CRF is not completely relieved by rest, unlike normal fatigue experienced day-to-day (2). Reported prevalence rates vary considerably and are dependent on cancer type, severity and treatment stage (2–6). Patients with advanced cancer are also known to experience more fatigue than patients with earlier stage disease (7).

It is important to recognise and manage CRF. However, a lack of agreed diagnostic criteria, variability across international guidelines, inconsistency in recognition and the existence of multiple scales to quantify fatigue all contribute to the complexity of effective management (3, 4, 8, 9). The available options can be broadly grouped into exercise interventions, psychosocial and pharmacological therapies.

## Exercise interventions

### In early-stage cancer and post-cancer therapy

It has long been known that physical exercise is beneficial for the management of cancer-related fatigue (CRF) in patients with solid tumours (10). In the last decade, several randomised controlled trials (RCTs) in cancer patients have demonstrated the effects of exercise interventions in alleviating fatigue in patients both during and after cancer therapy (11–14). Physical exercise appears to be safe and is recommended in cancer patients during active treatment with curative intent and in patients with lung cancer undergoing surgery (15). However, the role of exercise as an intervention for fatigue in advanced cancer is more ambiguous.

### In advanced cancer

#### Safety

An obvious concern is that whilst exercise may be safe and feasible for many cancer patients, in patients with advanced disease, there may be an increased risk of adverse events (e.g. fracture of skeletal metastases) or decreased scope for improvement (due to poor functional status precluding participation in exercise programmes). However, individual studies (16–18), systematic reviews (19•) and clinical guidelines (15) conclude or recommend that exercise is generally a safe and feasible intervention even in this population.

#### Efficacy

Evidence of effectiveness for exercise in patients with advanced cancer is more equivocal. One recent systematic review of studies involving heterogeneous populations with advanced solid tumour cancers did not find any significant or clinical fatigue improvements with exercise (20). In contrast, a more recent small meta-analysis (19•) of two RCTs, specific to patients with advanced cancer (16, 17), showed a significant effect of exercise (standardised mean difference (SMD) 0.31, 95% confidence interval [CI] 0.07–0.55).

Since the publication of those systematic reviews, several studies evaluating the efficacy of exercise for fatigue in advanced cancer have been published (18, 21–27). These studies have based their interventions on different types of physical exercise, the majority using a scheme consisting of a supervised first session, followed by a regimen of home-based activities. The exercise programmes have varied from 4- to 14-week duration, either using resistance or aerobic exercises, very frequently including walking. Some have used technologies to instruct or monitor exercise, from simple pedometers to more sophisticated blends of exercise, technology and gaming. However, most have been feasibility studies, with small sample sizes, underpowered to detect statistical significance, and in which the main outcome has not been improvement in fatigue per se, but rather mobility, ability to perform daily activities, quality-of-life or the safety of the intervention. Results have been diverse, with clinical fatigue improvements only statistically significant in two trials that were adequately powered for efficacy (25, 26). Wilkie and colleagues (26•) undertook an RCT, which included a population (*n* = 279) 60% of whom had advanced cancer (stages III and IV). They found that a 4-week tablet computer-based education programme resulted in significantly lower fatigue intensity in the intervention group (although interestingly there was no reported difference in activity levels between treatment arms). Navigante and colleagues (25) undertook a prospective randomised study in advanced gastrointestinal cancer patients (*n* = 64) at high risk of developing fatigue and reported that the exercise intervention reduced fatigue intensity (*p* = 0.019).

A review of five national and international guidelines on cancer-related fatigue (specifically focussing on those recommendations related to patients with advanced disease) concluded that there was moderate evidence that exercise activity should be recommended (28).

#### Duration

Whereas in patients with localised disease, relatively short duration (≤ 12 weeks) supervised exercise programmes show greater effects than longer or unsupervised interventions (29••), in patients with metastatic bone disease undergoing radiotherapy, the beneficial effects of exercise on fatigue may take 6 months to manifest (8).

#### Evidence for effectiveness of exercise as part of a multi-modal intervention

Two recent small studies used a combination of physical exercise and pharmacological therapy, in patients with advanced cancer and fatigue at baseline. In one non-randomised, preliminary study (*n* = 45), the combination of anamorelin, physical exercise and nutrition counselling for 6 weeks were associated with improvements in CRF (30). In the second (phase II) trial, participants (*n* = 76) were randomised to standardised physical activity for 4 weeks with either 4 mg or 8 mg of dexamethasone twice a day for 7 days (31). Both combination therapies resulted in an improvement of CRF that was maintained over 3 weeks and was similar with both high-dose and low-dose dexamethasone.

## Psychological interventions

Higher levels of cancer-related fatigue (CRF) are associated with psychosocial factors such as anxiety, depression and catastrophising thinking style (32, 33). These factors suggest that CRF is a complex experience, felt not only as a physical symptom but also through its effects on psychosocial functioning and quality-of-life. This all suggests that psychological approaches may be beneficial in the management of CRF.

### In cancer patients on treatment and in cancer survivors

#### Cognitive-behavioural approaches

A cognitive-behavioural therapy (CBT) model of CRF conceptualises the persistence of fatigue in cancer patients as an interplay between physical symptoms, emotions, thoughts and behaviours (34). A systematic review of psychological approaches for the management of fatigue in cancer survivors found significant treatment effects for all CBT interventions (35•).

Corbett and co-workers (36) conducted a systematic review, focusing on the psychological management of CRF in cancer survivors. They identified 33 RCTs investigating a variety of different psychological approaches to fatigue management. The studies were too heterogeneous to undertake a meta-analysis. Nonetheless, they found some evidence in support of CBT-based and mindfulness-based approaches, with more mixed evidence for the ‘other’ approaches they explored (e.g. reductions in CRF not being maintained at follow-up for health coaching and lifestyle interventions).

#### Psycho-educational approaches

Dolbeault and co-workers (37) undertook an RCT in 203 women after primary treatment for breast cancer. The intervention consisted of weekly 2-h sessions of a psycho-educational programme informed by CBT principles. The primary outcomes were state and trait anxiety, which showed an improvement. However, the authors also reported improvements in a number of secondary outcomes, including fatigue. This finding is supported by the results of Pearson and co-workers’ (9) scoping review, which evaluated nine broad approaches to managing CRF, including psycho-education (talking-based therapies and education) and supportive-expressive therapies. They identified 41 RCTs with a psycho-educational component, 25 (61%) of which reported statistically significant improvements in fatigue.

#### Other approaches

In contrast to the evidence in support of psycho-educational approaches, Pearson’s scoping review (9) found three RCTs involving expressive therapies, only one of which showed statistically significant improvement (a study of expressive writing).

Mindfulness-based interventions have become increasingly studied as a management strategy for CRF across cancer care (38, 39). These approaches include Mindfulness-Based Stress Reduction (MBSR) and Acceptance and Commitment Therapy (ACT). In this paradigm, the focus of change is not so much on reducing the symptom itself, but on reducing symptom-related suffering (40). Corbett’s systematic review of psychological approaches in CRF in cancer survivors (36) found mixed evidence for mindfulness-based approaches, with four of six studies showing significant improvements in CRF.

### In advanced cancer patients

Although there may be some benefit for psychological interventions as treatments for CRF in patients with earlier stage disease, the picture is less clear in patients with advanced cancers. In this population, CRF may differ in fundamental ways from the fatigue experienced by those with earlier stage disease. Mustian and colleagues’ meta-analysis (35•) of various fatigue treatments across the cancer spectrum suggested that patients with metastatic cancers report the least benefits. Furthermore, non-acceptance of having incurable cancer has also been linked with fatigue severity (32). It may be, for instance, that those struggling with CRF in advanced cancers are a specific cohort with distinct characteristics.

Several systematic reviews have concluded that the evidence for psychological interventions for fatigue in patients with advanced cancer is limited and of poor quality. Poort and co-workers (41) undertook a systematic review of RCTs of psychological interventions for fatigue in incurable adult cancer patients receiving treatment with palliative intent. From 14 studies they were able to extract data on 12 studies (*n* = 535 participants) for a meta-analysis. They found no evidence of benefit for heterogenous psychological interventions immediately after the intervention or at second follow-up and only poor-quality evidence in favour of psychological therapies at the first follow-up. Beatty and colleagues’ (42) review included RCTs evaluating psychological interventions for women with metastatic breast cancer (not specifically focussed on fatigue). They found five RCTs which reported on fatigue as an outcome, only one of which found a benefit (and in that trial fatigue had not been the primary outcome of interest) (43). Li and colleagues (44) reviewed RCTs of health-related outcomes of ACT for patients with advanced cancer. Across the four studies which had evaluated fatigue as an outcome, there was a non-significant improvement. In the light of the lack of high-quality evidence from these systematic reviews, Chapman and colleagues’ (28) practice review recommended that psychological therapies should not be used as a treatment for fatigue in patients with advanced cancer.

Despite Chapman and colleague’s negative appraisal of the evidence, there have been some more recent studies which have suggested that psychological therapies may help with CRF, even in patients with advanced cancer. For instance, one study (which was not included in the aforementioned systematic reviews), compared CBT to graded exercise therapy (GET) and treatment as usual (TAU) (22). Participants in the CBT arm attended a maximum of ten individual, 1-h sessions across 12 weeks. The authors reported that, at a 14-week follow-up, CBT significantly reduced CRF when compared with usual care, whilst this difference was not significant between the GET and TAU groups. Similarly, despite the inconclusive findings of their previous systematic review (44), Li and colleagues (45) undertook a single-blind RCT pilot study in 40 patients with advanced lung cancer and reported that ACT was feasible, acceptable and showed statistically significant improvements in CRF (but not in fatigue interference—the primary outcome). Thus, the role of ACT for treating CRF in advanced cancer still needs to be defined.

Whilst symptom reduction has commonly been the primary outcome used to evaluate effectiveness, third wave therapies (e.g. ACT; mindfulness-based interventions) place emphasis on symptom-related distress. In this instance, one may not necessarily observe changes in levels of fatigue but may instead observe changes in how patients relate to their fatigue or how fatigue contributes to their functional impairment. It is likely that placing increasing clinical and research salience on measures of quality-of-life, anxiety and depression alongside specific CRF measures will further add to the evolving picture of effectiveness of psychological therapies in the management of CRF.

## Pharmacological management

Numerous studies have been conducted investigating various pharmacological agents and their ability to reduce CRF, ranging from psychostimulants to corticosteroids, and nutritional supplements to hormonal agents. Despite this, evidence for any one of these agents remains limited and weak, usually due to a high heterogeneity between studies and an underrepresentation of advanced cancer patients (9), making recommendations for treating CRF difficult to establish. Another problem is that there has previously been a large placebo effect noted in cancer fatigue studies (46), which may make it more difficult to show the added superiority or otherwise of specific pharmacological therapies in randomised controlled trials. An interesting approach to tackling this issue has been described by Yennurajalingam and colleagues (47). In a recent trial of open-label placebo for fatigue in advanced cancer patients (in comparison to a waiting list control), the authors reported that open-label placebo significantly reduced fatigue after one week (with the effect being maintained for 4 weeks). Further understanding about how to maximise the placebo response in clinical practice (and to account for the effect in clinical trials) is warranted.

### Management of reversible comorbidities

Comorbid conditions, such as anaemia and pain, have been shown to contribute to the severity of fatigue and careful assessment and management of these may prove beneficial (2, 3, 48–50). A randomised control trial (RCT) in which cancer patients were assigned to receive different opioids, titrated to a dose that achieved optimum pain control, reported statistically significant reduction in levels of fatigue as a secondary outcome (51). In another RCT, patients were assigned to one of two groups; standard care vs protocolised patient-tailored treatment. The latter involved four nurse-led appointments during which nine physical symptoms—excluding fatigue—were discussed, assessed and treated (49). Results showed a significant reduction in self-reported fatigue, but no difference in quality-of-life measurements in the intervention group (49).

Erythropoiesis stimulating agents such as erythropoietin or darbopoetin have in the past mainly been used to treat anaemia caused by conditions such as chronic renal failure. In cancer patients, systematic reviews have reported that these agents, although improving quality-of-life and fatigue, are associated with increased risk of thromboembolic events and death (52–54). Their use must therefore be carefully balanced against any risk of adverse events.

### Herbal remedies and nutritional supplements

#### Ginseng

One of the most studied herbal remedies for CRF is ginseng, a commonly used ingredient in Chinese medicine (55). The active ingredients in ginseng are ginsenosides which are hypothesised to improve CRF through anti-inflammatory and central nervous system stimulant effects (56). Eleven types of ginseng exist, with American ginseng (Panax quinquefolius), Asian ginseng (Panax ginseng) and Korean ginseng (Ginseng radix) being the most commonly studied varieties (56). One large (*n* = 364), well-conducted RCT compared American ginseng to placebo in cancer patients undergoing (or having undergone) treatment with curative intent. The primary outcome was fatigue after 4-week treatment, with planned analyses at 8-week and sub-group analyses according to whether patients were on treatment or post-treatment. In the combined population, there was no improvement in fatigue at 4 weeks, but a significant improvement by 8 weeks. On sub-group analysis, ginseng improved fatigue in patients receiving cancer treatment at both time points, but did not improve fatigue in post-treatment patients at either time point. Yennurajalingam and colleagues (57) evaluated the effects of Asian ginseng on patients with advanced cancer and fatigue. In a double-blind, randomised placebo-controlled trial (*n* = 127), they were unable to find any superiority for ginseng over placebo after either 2 weeks or 4 weeks of therapy. A subsequent systematic review and meta-analysis (58•) of four RCTs (including both the Barton and Yennurajalingham studies described above) concluded that there was weak evidence for benefit of ginseng over placebo (standard mean difference [SMD], −0.21; 95%; confidence interval [CI], −0.42 to 0.00).

#### Guarana

Guarana is a plant found in the Amazonian rainforest of which extracts have been evaluated for their potential benefits in CRF. A recent systematic review (59) identified seven RCTs which have evaluated guarana versus placebo in a variety of different tumour types, across all stages (although predominantly in patients with non-metastatic disease). Of the seven trials, four showed no benefit for guarana. A meta-analysis was undertaken for the three trials which had been undertaken in patients with breast cancer, and this also found no benefit for the intervention.

#### Mistletoe

Mistletoe is a parasitic plant with white berries and with a long history of use as a complementary and/or alternative medicine by patients with cancer. It has been evaluated as a treatment for CRF in a number of studies. A recent systematic review (60) of studies undertaken in patients with a variety of different cancers and cancer stages (predominantly non-metastatic) identified 12 RCTs (two of which were double-blind). On meta-analysis, there was a significant benefit for the intervention over placebo (SMD –0.48 [95% CI –0.82 to –0.14]; *p* = 0.006). However, the results need to be interpreted with caution, due to a high risk of bias for 11 of the 12 included studies, which also demonstrated a high degree of heterogeneity. Furthermore, another (smaller) systematic review (61), evaluating the effects of mistletoe extract on various aspects of quality-of-life in heterogenous cancer patients (also mostly non-metastatic), failed to find a statistically significant effect of the intervention on fatigue. The nine trials identified by this review were all unblinded and there was high degree of heterogeneity and risk of bias.

#### Other herbal/nutritional remedies

Overall, whilst nutrition plays an important role in the health and well-being of cancer patients, there is insufficient evidence to make definitive dietary recommendations about the treatment of CRF (62). There is insufficient evidence to recommend other nutritional supplements such as co-enzyme Q10, astragalus, or l-carnitine (63).

### Psychostimulants

Psychostimulants, such as methylphenidate, dexamphetamine and modafinil are among the most widely studied pharmacological treatments for CRF. There have been numerous systematic reviews and meta-analyses to evaluate the effects of these medications on CRF in heterogenous cancer patient groups (from early through to advanced stage in patients on or off active treatment) (35, 64) or more focused only on those with advanced disease (65).

#### Methylphenidate

Belloni and colleagues (66••) have undertaken a systematic review of systematic reviews evaluating pharmacological therapies for CRF. They identified six systematic reviews (published between January 2010 to July 2020), which summarised the results of 70 primary studies involving 6203 participants. Trials ranged from less than 1 to 12 weeks in length and included studies evaluating various psychostimulants (most commonly methylphenidate), with a variety of dosing schedules. Across eight meta-analyses conducted in six systematic reviews, Belloni reported a moderate and statistically significant benefit for psychostimulants in general (SMD = −0.20 [95% CI: −0.33 to −0.08; *p* < 0.01]) and for methylphenidate in particular (SMD = −0.48 [95 % CI: −0.75 to −0.27; *p* < 0.01]). Furthermore, in the two meta-analyses which evaluated adverse effects, there was no statistically significant differences between treatment and control arms. This is important because the potential adverse effects of psychostimulants include symptoms such as nausea, insomnia, anxiety and anorexia, which may be particularly troubling when the treatment is being used as a palliative measure for patients with advanced disease.

Since the publication of Belloni’s review of reviews, two more papers have been published examining the effects of methylphenidate on CRF in patients with advanced cancer. Pederson and colleagues (67) undertook a small (*n* = 28), short-term (1 week), double-blind placebo-controlled trial of “as required” methylphenidate versus placebo and reported a significant reduction in fatigue at 2 and 5 h post-dose. Centeno and colleagues (68) reported the results of a double-blind, placebo-controlled, 6-day trial of methylphenidate (15 to 35 mg/day individually titrated) also in patients (*n* = 77) with advanced cancer. Unfortunately, the study failed to recruit the desired sample size and was underpowered. Nonetheless, the authors reported no benefit for methylphenidate over placebo.

#### Modafanil

Jean-Pierre and colleagues (69) evaluated modafinil (a vigilance promoting agent) in 631 ambulatory cancer patients receiving chemotherapy. The study found no benefit for fatigue in the study population as a whole; however, in a planned sub-group analysis of the 458 participants with severe fatigue, there was a small but statistically significant improvement in fatigue. Spathis and colleagues (70) evaluated the effect of modafinil in “off treatment” patients (*n* = 160) with non-small cell lung cancer and reported no benefit for modafinil over placebo, even in the sub-set of participants with severe fatigue. Although it should be noted that in this study, the sub-group analysis of patients with severe fatigue was not pre-planned and the number of patients with severe fatigue was small.

### Corticosteroids

Corticosteroids have long been supposed to have non-specific benefits for symptom control, mood and quality-of-life in patients with advanced cancer (71), but there has been limited research to specifically evaluate their role specifically for the relief of CRF. Yennurajalingam and colleagues (72••) undertook a double-blind randomised placebo controlled of dexamethasone 4 mg/day in patients with advanced cancer who were experiencing multiple symptoms (three of more). There was an improvement in the primary outcome (reduction in fatigue) after 14 days. However, given the known adverse effects of steroids, their use would currently appear to be limited to palliative and end-of-life care settings only, at least until further research is undertaken to evaluate other specific circumstances in which the benefit/burden ratio may be in favour of their use.

### Other agents

A variety of other agents have been investigated for their role in reducing CRF including paroxetine, bupropion, donepezil, thyrotropin-releasing hormone, melatonin, testosterone and ATP. None of these can be recommended due to a lack of good quality evidence of benefit (6, 8, 50, 73, 74).

## Guideline recommendations

Various professional bodies and organisations have developed guidelines for the management of cancer-related fatigue (1, 75, 76). Across the various guidelines, there is some consensus about the importance of identifying and evaluating fatigue and addressing reversible comorbidities (48, 76–79). The most up-to-date guidance emanates from the National Comprehensive Cancer Network (NCCN), which is annually updated (1), and from the European Society of Medical Oncology (ESMO) (76).

The NCCN guidelines make a number of firm (category 1 - high-level evidence, uniform consensus) recommendations in favour of physical activity, yoga, CBT and psycho-educational approaches in patients on and post-treatment. In addition, NCCN guidelines recommend massage in patients on treatment and mindfulness-based stress reduction, supportive-expressive therapies and CBT for insomnia in patients post-treatment. Although providing tailored guidance on interventions for fatigue for end-of-life care, the NCCN guidelines make no firm (category 1) recommendations about specific interventions in this population.

None of the ESMO recommendations reached the highest strength (indicating both strong evidence of efficacy and substantial clinical benefit to support recommendation for use) but several recommendations were regarded as reaching category B (moderate evidence of efficacy—or strong evidence of efficacy but only limited clinical benefit—supports recommendation for use). Those management strategies were physical activity, psycho-educational approaches and CBT. No consensus was reached about whether or not to recommend methylphenidate, mistletoe or ginseng. The only pharmacological approach to be recommended was dexamethasone in patients with advanced cancer. Several drugs or nutritional supplements received a recommendation not to use: modafinil, paroxetine; donepezil, guarana, l-carnitine, co-enzyme Q10 and astragalus.

## Conclusions

Fatigue is a common and distressing symptom experienced by patients with cancer that can be found at all stages of the illness trajectory, with higher prevalence in patients with more advanced disease. CRF can have profound effects on quality-of-life and physical functioning (80). There is growing evidence that physical activity and psychological therapies (particularly CBT and psycho-educational approaches) may be of benefit in patients with earlier stage disease, undergoing active treatment and in disease-free cancer survivors. There is less strong evidence for pharmacological interventions. There are reasons to believe that interventions that are feasible, safe or effective in patients with earlier stage disease may not be so in patients with more advanced disease. For example, it is conceivable that exercise may be safe and effective in patients undergoing potentially curative chemotherapy or radiotherapy, but may carry unacceptable risks or be infeasibly burdensome in patients with advanced cancer. Furthermore, even if exercise or CBT were found to be effective in earlier stage disease, the mechanisms or maintenance of fatigue in patients with advanced disease may be appreciably different, such that these interventions are no longer efficacious.

There is some weak evidence that in advanced disease exercise may be beneficial for the relief of cancer fatigue, but further studies are needed and it is likely that specifically tailored exercise programmes more appropriate for this population should be developed and evaluated. Similarly, there is only weak evidence for the benefits of psychological therapies to relieve fatigue in this group with the best evidence for CBT. No pharmacological therapies have good evidence for benefit in patients with advanced disease and relatively few CRF trials have specifically targeted this population. In patients towards the end-of-life, there is some evidence in favour of short-term use of dexamethasone, but other interventions (e.g. methylphenidate) require further evaluation before they can be recommended for general use.

## References

[1.••] Jankowski C, Berger A, Aranha O, Banerjee C, Breitbart W, Carpenter K, et al. NCCN Guidelines Version 2.2022 Cancer-Related Fatigue. NCCN. 2022;https://www.nccn.org/. Evidence-based guidelines that are updated regularly and relate to patients on and off treatment and in patients at the end-of-life.

[2] Soones T, Ombres R, Escalante C (2022). An update on cancer-related fatigue in older adults: a narrative review. J Geriatr Oncol..

[3.•] Chapman EJ, Martino ED, Edwards Z, Black K, Maddocks M, Bennett MI. Practice review: evidence-based and effective management of fatigue in patients with advanced cancer. Palliat Med. 2022;36(1):7–14 Pragmatic guideline that gives short and general recommendations for the management of fatigue in patients with advanced cancer. 10.1177/02692163211046754 PMC8793304 34903113

[4] Henson LA, Maddocks M, Evans C, Davidson M, Hicks S, Higginson IJ (2020). Palliative care and the management of common distressing symptoms in advanced cancer: pain, breathlessness, nausea and vomiting, and fatigue. J Clin Oncol..

[5] Maqbali MA (2021). Cancer-related fatigue: an overview. British Journal of Nursing..

[6] Thong MSY, van Noorden CJF, Steindorf K, Arndt V. Cancer-related fatigue: causes and current treatment options. Curr Treat Options Oncol. 2020;21(2):17. 10.1007/s11864-020-0707-5 PMC8660748 32025928

[7] Al Maqbali M, Al Sinani M, Al Naamani Z, Al Badi K, Tanash MI (2021). Prevalence of fatigue in patients with cancer: a systematic review and meta-analysis. J Pain Symptom Manage..

[8] Mücke M, Mochamat., Cuhls H, Peuckmann-Post V, Minton O, Stone P, et al. Pharmacological treatments for fatigue associated with palliative care (Review). Cochrane Database of Systematic Reviews. 2015(5). 10.1002/14651858.CD006788.pub3 PMC6483317 26026155

[9] Pearson EJM, Morris ME, di Stefano M, McKinstry CE. Interventions for cancer-related fatigue: a scoping review. Eur J Cancer Care (Engl). 2018;27(1). 10.1111/ecc.12516 27254272

[10] Cramp F, Byron-Daniel J (2012). Exercise for the management of cancer-related fatigue in adults. Cochrane Database Syst Rev..

[11] Gerritsen JK, Vincent AJ (2016). Exercise improves quality of life in patients with cancer: a systematic review and meta-analysis of randomised controlled trials. BJSM online..

[12] Mishra SI, Scherer RW, Geigle PM, Berlanstein DR, Topaloglu O, Gotay CC (2012). Exercise interventions on health-related quality of life for cancer survivors. Cochrane Database Syst Rev..

[13] Mishra SI, Scherer RW, Snyder C, Geigle PM, Berlanstein DR, Topaloglu O (2012). Exercise interventions on health-related quality of life for people with cancer during active treatment. Cochrane Database Syst Rev..

[14] Cormie P, Zopf EM, Zhang X, Schmitz KH (2017). The Impact of Exercise on Cancer Mortality, Recurrence, and Treatment-Related Adverse Effects. Epidemiol Rev..

[15] Ligibel JA, Bohlke K, May AM, Clinton SK, Demark-Wahnefried W, Gilchrist SC, Irwin ML, Late M, Mansfield S, Marshall TF, Meyerhardt JA, Thomson CA, Wood WA, Alfano CM (2022). Exercise, diet, and weight management during cancer treatment: ASCO guideline. J Clin Oncol..

[16] Jensen W, Baumann FT, Stein A, Bloch W, Bokemeyer C, de Wit M, Oechsle K (2014). Exercise training in patients with advanced gastrointestinal cancer undergoing palliative chemotherapy: a pilot study. Support Care Cancer..

[17] Adamsen L, Quist M, Midtgaard J, Andersen C, Moller T, Knutsen L (2006). The effect of a multidimensional exercise intervention on physical capacity, well-being and quality of life in cancer patients undergoing chemotherapy. Support Care Cancer..

[18] Yee J, Davis GM, Hackett D, Beith JM, Wilcken N, Currow D, Emery J, Phillips J, Martin A, Hui R, Harrison M, Segelov E, Kilbreath SL (2019). Physical activity for symptom management in women with metastatic breast cancer: a randomized feasibility trial on physical activity and breast metastases. J Pain Symptom Manage..

[19] Mochamat, Cuhls H, Sellin J, Conrad R, Radbruch L, Mucke M (2021). Fatigue in advanced disease associated with palliative care: a systematic review of non-pharmacological treatments. Palliat Med..

[20] Nadler MB, Desnoyers A, Langelier DM, Amir E (2019). The effect of exercise on quality of life, fatigue, physical function, and safety in advanced solid tumor cancers: a meta-analysis of randomized control trials. J Pain Symptom Manage..

[21] Villumsen BR, Jorgensen MG, Frystyk J, Hordam B, Borre M (2019). Home-based ‘exergaming’ was safe and significantly improved 6-min walking distance in patients with prostate cancer: a single-blinded randomised controlled trial. BJU Int..

[22] Poort H, Peters M, van der Graaf WTA, Nieuwkerk PT, van de Wouw AJ (2020). Nijhuis-van der Sanden MWG, et al. Cognitive behavioral therapy or graded exercise therapy compared with usual care for severe fatigue in patients with advanced cancer during treatment: a randomized controlled trial. Ann Oncol..

[23] Frikkel J, Beckmann M, De Lazzari N, Gotte M, Kasper S, Hense J (2021). Changes in fatigue, barriers, and predictors towards physical activity in advanced cancer patients over a period of 12 months-a comparative study. Support Care Cancer..

[24] Cheung DST, Takemura N, Lam TC, Ho JCM, Deng W, Smith R, Yan Y, Lee AWM, Lin CC (2021). Feasibility of aerobic exercise and Tai-Chi interventions in advanced lung cancer patients: a randomized controlled trial. Integ Cancer Ther..

[25] Navigante A, Cresta Morgado P, Daud ML, Dos Santos RH, Kolberg M, Marazzi C (2022). Physical exercise and fatigue in advanced gastrointestinal cancer during chemotherapy. BMJ supportive & palliative care..

[26] Wilkie DJ, Schwartz AL, Liao WC, Fullwood D, Wu Y, Farquharson TW (2022). Reduced cancer-related fatigue after tablet-based exercise education for patients. Cancer Control..

[27] Ester M, Culos-Reed SN, Abdul-Razzak A, Daun JT, Duchek D, Francis G, Bebb G, Black J, Arlain A, Gillis C, Galloway L, Capozzi LC (2021). Feasibility of a multimodal exercise, nutrition, and palliative care intervention in advanced lung cancer. BMC Cancer..

[28] Chapman EJ, Martino ED, Edwards Z, Black K, Maddocks M, Bennett MI (2022). Practice review: evidence-based and effective management of fatigue in patients with advanced cancer. Palliat Med..

[29] Van Vulpen JK, Sweegers MG, Peeters PHM, Courneya KS, Newton RU, Aaronson NK (2020). Moderators of exercise effects on cancer-related fatigue: a meta-analysis of individual patient data. Med Sci Sports Exerc.

[30] Yennurajalingam S, Basen-Engquist K, Reuben JM, Fellman BM, Shete S, Maddi R, Williams JL, Dev R, Hui D, Bruera E (2022). Anamorelin combined with physical activity, and nutritional counseling for cancer-related fatigue: a preliminary study. Support Care Cancer..

[31] Yennurajalingam S, Valero V, Lu Z, Liu DD, Busaidy NL, Reuben JM, et al. Combination therapy of physical activity and dexamethasone for cancer-related fatigue: a phase II randomized double-blind controlled trial. J. 2021:1-9. 10.6004/jnccn.2021.7066 34965510

[32] Peters ME, Goedendorp MM, Verhagen CA, Bleijenberg G, van der Graaf WT (2016). Fatigue and its associated psychosocial factors in cancer patients on active palliative treatment measured over time. Support Care Cancer..

[33] Hughes A, Suleman S, Rimes KA, Marsden J, Chalder T (2020). Cancer-related fatigue and functional impairment - towards an understanding of cognitive and behavioural factors. J Psychosom Res..

[34] Gielissen MF, Verhagen S, Witjes F, Bleijenberg G (2006). Effects of cognitive behavior therapy in severely fatigued disease-free cancer patients compared with patients waiting for cognitive behavior therapy: a randomized controlled trial. J Clin Oncol..

[35] Mustian KM, Alfano CM, Heckler C, Kleckner AS, Kleckner IR, Leach CR (2017). Comparison of pharmaceutical, psychological, and exercise treatments for cancer-related fatigue: a meta-analysis. JAMA Oncol..

[36] Corbett TK, Groarke A, Devane D, Carr E, Walsh JC, McGuire BE (2019). The effectiveness of psychological interventions for fatigue in cancer survivors: systematic review of randomised controlled trials. Syst..

[37] Dolbeault S, Cayrou S, Bredart A, Viala AL, Desclaux B, Saltel P (2009). The effectiveness of a psycho-educational group after early-stage breast cancer treatment: results of a randomized French study. Psychooncology..

[38] Hulbert-Williams NJ, Storey L, Wilson KG (2015). Psychological interventions for patients with cancer: psychological flexibility and the potential utility of Acceptance and Commitment Therapy. Eur J Cancer Care (Engl).

[39] Zimmermann FF, Burrell B, Jordan J (2018). The acceptability and potential benefits of mindfulness-based interventions in improving psychological well-being for adults with advanced cancer: a systematic review. Complement Ther Clin Pract.

[40] Mosher CE, Secinti E, Li R, Hirsh AT, Bricker J, Miller KD, Schneider B, Storniolo AM, Mina L, Newton EV, Champion VL, Johns SA (2018). Acceptance and commitment therapy for symptom interference in metastatic breast cancer patients: a pilot randomized trial. Support Care Cancer..

[41] Poort H, Peters M, Bleijenberg G, Gielissen MF, Goedendorp MM, Jacobsen P (2017). Psychosocial interventions for fatigue during cancer treatment with palliative intent. Cochrane Database Syst Rev..

[42] Beatty L, Kemp E, Butow P, Girgis A, Schofield P, Turner J, Hulbert-Williams NJ, Levesque JV, Koczwara B (2018). A systematic review of psychotherapeutic interventions for women with metastatic breast cancer: context matters. Psychooncology..

[43] Spiegel D, Bloom JR, Yalom I (1981). Group support for patients with metastatic cancer. A randomized outcome study. Arch Gen Psychiatry..

[44] Li H, Wong CL, Jin X, Chen J, Chong YY, Bai Y (2021). Effects of acceptance and commitment therapy on health-related outcomes for patients with advanced cancer: a systematic review. Int J Nurs Stud..

[45] Li H, Jin X, Ng MSN, Mann KF, Wang N, Wong CL (2022). Effects of acceptance and commitment therapy on fatigue interference and health-related quality of life among patients with advanced lung cancer: a pilot randomized controlled trial. Asia-Pac..

[46] Roji R, Stone P, Ricciardi F, Candy B (2020). Placebo response in trials of drug treatments for cancer-related fatigue: a systematic review, meta-analysis and meta-regression. BMJ supportive & palliative care..

[47] Yennurajalingam S, Azhar A, Lu Z, Rodriguez AJ, Arechiga AB, Guerra-Sanchez M, Stanton P, Andersen CR, Urbauer DL, Bruera E (2022). Open-Label Placebo for the Treatment of Cancer-Related Fatigue in Patients with Advanced Cancer: A Randomized Controlled Trial. Oncologist..

[48] Berger A, Alvarez-Perez A, Carpenter KM, Cleeland C, Elsenberger MA, Jacobsen PB (2015). Cancer-Related Fatigue, Version 2.2015. Journal of the National Comprehensive Cancer Network..

[49] de Raaf PJ, de Klerk C, Timman R, Busschbach JJ, Oldenmenger WH, van der Rijt CC (2013). Systematic monitoring and treatment of physical symptoms to alleviate fatigue in patients with advanced cancer: a randomized controlled trial. J Clin Oncol..

[50] Ripamonti CI, Antonuzzo A, Bossi P, Cavalieri S, Roila F, Fatigoni S (2018). Fatigue, a major still underestimated issue. Curr Opin Oncol..

[51] Nosek K, Leppert W, Nosek H, Wordliczek J, Onichimowski D (2017). A comparison of oral controlled-release morphine and oxycodone with transdermal formulations of buprenorphine and fentanyl in the treatment of severe pain in cancer patients. Drug Des Devel Ther..

[52] Tomlinson D, Robinson PD, Oberoi S, Cataudella D, Culos-Reed N, Davis H, Duong N, Gibson F, Götte M, Hinds P, Nijhof SL, van der Torre P, Cabral S, Dupuis LL, Sung L (2018). Pharmacologic interventions for fatigue in cancer and transplantation: a meta-analysis. Curr Oncol..

[53] Tonia T, Mettler A, Robert N, Schwarzer G, Seidenfeld J, Weingart O, Hyde C, Engert A, Bohlius J, Cochrane Haematological Malignancies Group (2012). Erythropoietin or darbepoetin for patients with cancer. Cochrane Database Syst Rev..

[54] Minton O, Richardson A, Sharpe M, Hotopf M, Stone P (2008). A systematic review and meta-analysis of the pharmacological treatment of cancer-related fatigue. J Natl Cancer Inst..

[55] Inglis JE, Lin PJ, Kerns SL, Kleckner IR, Kleckner AS, Castillo DA, Mustian KM, Peppone LJ (2019). Nutritional Interventions for Treating Cancer-Related Fatigue: A Qualitative Review. Nutr Cancer..

[56] Sadeghian M, Rahmani S, Zendehdel M, Hosseini SA, Zare JA (2021). Ginseng and Cancer-Related Fatigue: A Systematic Review of Clinical Trials. Nutr Cancer..

[57] Yennurajalingam S, Tannir NM, Williams JL, Lu Z, Hess KR, Frisbee-Hume S (2017). A double-blind, randomized, placebo-controlled trial of Panax ginseng for cancer-related fatigue in patients with advanced cancer. J National Compre Cancer Net.

[58] Luo WT, Huang TW (2022). Effects of ginseng on cancer-related fatigue: a systematic review and meta-analysis of randomized controlled trials. Cancer Nurs.

[59] de Araujo DP, Pereira P, Fontes AJC, Marques KDS, de Moraes EB, Guerra RNM (2021). The use of guarana (Paullinia cupana) as a dietary supplement for fatigue in cancer patients: a systematic review with a meta-analysis. Support Care Cancer..

[60] Pelzer F, Loef M, Martin DD, Baumgartner S (2022). Cancer-related fatigue in patients treated with mistletoe extracts: a systematic review and meta-analysis. Support Care Cancer..

[61] Loef M, Walach H (2020). Quality of life in cancer patients treated with mistletoe: a systematic review and meta-analysis. BMC Complement Med Ther..

[62] Baguley BJ, Skinner TL, Wright ORL (2019). Nutrition therapy for the management of cancer-related fatigue and quality of life: a systematic review and meta-analysis. Br J Nutr..

[63] Ripamonti CI, Antonuzzo A, Bossi P, Cavalieri S, Roila F, Fatigoni S (2018). Fatigue, a major still underestimated issue. [Review]. Curr Opin Oncol..

[64] Minton O, Richardson A, Sharpe M, Hotopf M, Stone P (2010). Drug therapy for the management of cancer-related fatigue. Cochrane Database Syst Rev..

[65] Mucke M, Mochamat, Cuhls H, Peuckmann-Post V, Minton O, Stone P (2015). harmacological treatments for fatigue associated with palliative care. [Review]. Cochrane Database Syst Rev.

[66] Belloni S, Arrigoni C, de Sanctis R, Arcidiacono MA, Dellafiore F, Caruso R (2021). A systematic review of systematic reviews and pooled meta-analysis on pharmacological interventions to improve cancer-related fatigue. Crit Rev Oncol Hematol..

[67] Pedersen L, Lund L, Petersen MA, Sjogren P, Groenvold M (2020). Methylphenidate as Needed for Fatigue in Patients With Advanced Cancer. A Prospective, Double-Blind, and Placebo-Controlled Study. J Pain Symptom Manage..

[68] Centeno C, Roji R, Portela MA, De Santiago A, Cuervo MA, Ramos D (2022). Improved cancer-related fatigue in a randomised clinical trial: methylphenidate no better than placebo. BMJ supportive & palliative care..

[69] Jean-Pierre P, Morrow GR, Roscoe JA, Heckler C, Mohile S, Janelsins M, Peppone L, Hemstad A, Esparaz BT, Hopkins JO (2010). A phase 3 randomized, placebo-controlled, double-blind, clinical trial of the effect of modafinil on cancer-related fatigue among 631 patients receiving chemotherapy: a University of Rochester Cancer Center Community Clinical Oncology Program Research base study. Cancer..

[70] Spathis A, Fife K, Blackhall F, Dutton S, Bahadori R, Wharton R, O'Brien M, Stone P, Benepal T, Bates N, Wee B (2014). Modafinil for the treatment of fatigue in lung cancer: results of a placebo-controlled, double-blind, randomized trial. J Clin Oncol..

[71] Della Cuna GR, Pellegrini A, Piazzi M (1989). Effect of methylprednisolone sodium succinate on quality of life in preterminal cancer patients: a placebo-controlled, multicenter study. The Methylprednisolone Preterminal Cancer Study Group. Eur J Cancer Clin Oncol..

[72] Yennurajalingam S, Frisbee-Hume S, Palmer JL, Delgado-Guay MO, Bull J, Phan AT (2013). Reduction of cancer-related fatigue with dexamethasone: a double-blind, randomized, placebo-controlled trial in patients with advanced cancer. J Clin Oncol..

[73] Mohandas H, Jaganathan SK, Mani MP, Ayyar M, Rohini Thevi GV (2017). Cancer-related fatigue treatment: an overview. J Cancer Res Ther..

[74] Sun X, Chen Y, Cheung WK, Wu IX, Xiao F, Chung VC (2021). Pharmacological Interventions for the Management of Cancer-Related Fatigue Among Cancer Survivors: Systematic Review and Meta-Analysis. Integr Cancer Ther..

[75] Bower JE, Bak K, Berger A, Breitbart W, Escalante CP, Ganz PA, Schnipper HH, Lacchetti C, Ligibel JA, Lyman GH, Ogaily MS, Pirl WF, Jacobsen PB, American Society of Clinical Oncology (2014). Screening, assessment, and management of fatigue in adult survivors of cancer: an American Society of Clinical oncology clinical practice guideline adaptation. J Clin Oncol..

[76] Fabi A, Bhargava R, Fatigoni S, Guglielmo M, Horneber M, Roila F, Weis J, Jordan K, Ripamonti CI, ESMO Guidelines Committee. Electronic address: clinicalguidelines@esmo.org (2020). Cancer-related fatigue: ESMO Clinical Practice Guidelines for diagnosis and treatment. Ann Oncol..

[77] Bower JE (2014). Cancer-related fatigue--mechanisms, risk factors, and treatments. Nat Rev Clin Oncol..

[78] Howell D, Keshavarz H, Broadfield L, Hack T, Hamel M, Harth T, et al. 2015 A Pan Canadian Practice Guideline for Screening, Assessment, and Management of Cancer-Related Fatigue in Adults. Canadian Partnership Against Cancer (Cancer Journey Advisory Group) and the Canadian Association of Psychosocial Oncology. 1-252.

[79] Mitchell SA, Albrecht TA, Alkaiyat MO, Browne K, Clark JC, DeGennaro RM, et al. Fatigue - Oncology Nursing Society: Oncology Nurseing Society; 2017 [2017:[Available from: https://www.ons.org/pep/fatigue?display=pepnavigator&sort_by=created&items_per_page=50.

[80] Stone P, Ream E, Richardson A, Thomas H, Andrews P, Campbell P (2003). Cancer-related fatigue--a difference of opinion? Results of a multicentre survey of healthcare professionals, patients and caregivers. Eur J Cancer Care (Engl)..

